# Comparison of Verbal and Emotional Responses of Elderly People with Mild/Moderate Dementia and Those with Severe Dementia in Responses to Seal Robot, PARO

**DOI:** 10.3389/fnagi.2014.00257

**Published:** 2014-09-26

**Authors:** Kazue Takayanagi, Takahiro Kirita, Takanori Shibata

**Affiliations:** ^1^Seiwaen Geriatric Institute, Medical Cooperation Seiwakai, Ohsu, Japan; ^2^Medical Education Center, Nippon Medical School, Tokyo, Japan; ^3^Department of Psychology, Faculty of Social Welfare, Iwate Prefectural University, Takizawa, Japan; ^4^National Institute of Advanced Industrial Science and Technology (AIST), Tsukuba, Japan; ^5^Tokyo Institute of Technology, Yokohama, Japan; ^6^The AgeLab, Massachusetts Institute of Technology, Boston, MA, USA

**Keywords:** PARO, seal robot, psychological response, mild/moderate dementia, severe dementia

## Abstract

**Introduction:** The differences in verbal and emotional responses to a baby seal robot, PARO, of elderly people with dementia residing at an elderly nursing care facility were analyzed. There were two groups of elderly people: one was with mild/moderate dementia (M-group) that consisted with 19 elderly residents in the general ward, and the other was with severe dementia (S-group) that consisted with 11 elderly residents in the dementia ward.

**Method:** Each elderly resident in both groups interacted with either PARO or a control (stuffed lion toy: Lion) brought by a staff at each resident’s private room. Their responses were recorded on video. Behavioral analysis of the initial 6 min of the interaction was conducted using a time sampling method.

**Results:** In both groups, subjects talked more frequently to PARO than to Lion, showed more positive changes in emotional expression with PARO than with Lion, and laughed more frequently with PARO than with Lion. Subjects in M-group even showed more negative emotional expressions with Lion than with PARO. Furthermore, subjects in S-group showed neutral expression more frequently with Lion than with PARO, suggesting more active interaction with PARO. For subjects in M-group, frequencies of touching and stroking, frequencies of talking to staff member, and frequencies of talking initiated by staff member were significantly higher with Lion than with PARO.

**Conclusion:** The elderly people both with mild/moderate dementia and with severe dementia showed greater interest in PARO than in Lion. The results suggest that introducing PARO may increase willingness of the staff members to communicate and work with elderly people with dementia, especially those with mild/moderate dementia who express their demand of communication more than those with severe dementia.

## Introduction

Dementia is associated with behavioral and psychological disorders, and with increase of workload for professional care staffs in nursing homes.

**Figure 1:**
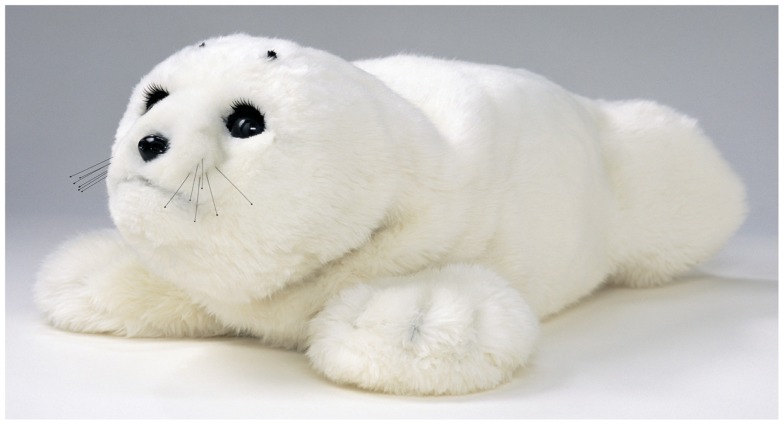
**Baby Seal Robot, PARO (Shibata, 2012)**.

The baby seal robot, PARO, is a neurological therapeutic medical device for non-pharmacological intervention (Figure 1) (Shibata, 2012). Demonstrative and clinical experiments showed PARO’s therapeutic effects, such as reduced stress, relief in depression, reduced anxiety, reduced pain, relaxation and suppression of behavioral and psychological symptoms related to dementia, and improved and recovered communication ability.

However, there are still lacks of evidences of its effects. Reasons for this are that if the responses of elderly subjects are examined, there are few parameters for comparative study depending on levels of dementia of elderly people; evaluation involves long-term observation, which may have problems of environmental bias or changes within subjects; and it is difficult to carry out psychological analysis (Moyle et al., 2013; Robinson et al., 2013a). Although psychological indices have been applied, there are few studies in which a psychologist has carried out a scientific analysis. Also, there is possibility of information bias or observer bias in research with demented elderly subjects (Robinson et al., 2013b). Regardless of such difficulties, PARO was compared to a living dog at a nursing home in randomized control trials (RCT), and the result showed PARO improved loneliness significantly (Robinson et al., 2013a). PARO was also compared to reading activity at a nursing home in RCT, and results showed reduction of anxiety, improvement of mood, and improvement of quality of life of elderly significantly (Moyle et al., 2013).

Here, we compared the effects of PARO to those of a stuffed lion toy (Lion) as a control on elderly residents with mild/moderate dementia (M-group) in the general ward and those with severe dementia (S-group) in the dementia ward at a nursing care facility.

The research questions were as follows:

1. What are the broad trends that can be grasped with respect to the effects of introducing PARO?
2. Does PARO have a psychological effect on residents with severe dementia in the dementia ward compared to residents with mild/moderate dementia in the general ward?
3. Can the introduction of PARO improve willingness of care staff members to work on interventions for elderly people with dementia?

The authors have obtained full informed consent from subjects and/or legal representatives of this study and got the approval from an ethical committee at Seiwakai Foundation.

## Materials and Methods

### Subjects

The subjects were 25 residents of the general ward (mean age; 84.9 + 9.1 years) and 11 residents of the dementia ward (mean age; 87.5 + 12.8 years) at our facility during the period from September 2011 to May 2012. The subjects and their families had written informed consent to participate in the study. Elderly residents with dementia are ranked into five levels (I, II, III, IV, and M) according to their needs, which includes ADL, physical and psychological condition, and those whose degrees of independence in everyday life (Japanese Ministry of Health, Labour and Welfare, 1993) are judged to be at level III or above are admitted to a dementia ward recognized under the care insurance system. Level III is defined as: “Since they have symptoms, behaviors and difficulties in understanding that make them difficult to live in their everyday life, they need care.”

Because of the limitations of elderly nursing care facilities, some of the residents were transferred to their own homes or to the special nursing homes for the elderly, or sometimes they were hospitalized due to the illness. Thus, the psychological evaluation was actually carried out on 19 residents in the general ward as the mild/moderate dementia group (M-group) and 11 in the dementia ward as the severe dementia group (S-group) in the period of trials.

As for the level of dementia of subjects in the groups, the S-group scored a mean of 8.8 points and the M-group scored a mean of 16.4 points on Hasegawa’s Dementia Scale (the maximum score was 30 points, and people with the scores below 20 were judged “demented”). Hasegawa’s Dementia Scale is similar to the MMSE and includes the following questions:

1. age, 2. disorientation of time and date, 3. disorientation of location, 4. mentioned immediate inscription of words, 5. calculation, 6. digit span backward, 7 delayed recall of words, 8. goods-mentioned inscription, 9. fluency of language.

The levels of communication of subjects were from fairly good to capable to respond to the questions.

### Methods

A staff member (always the same person) brought PARO or Lion, and stayed with the subject for 15 min in the subject’s private room. All subjects were exposed to both PARO and Lion, and the intervals between sessions were 3–6 months. The behavior of the resident was recorded on video.

The Ethics committee at Seiwakai Foundation approved this experimental study, and informed consent was obtained from all subjects and their families.

#### Subjects for the video analysis

The videos of residents who participated in both the experimental (PARO) and control (Lion) interaction conditions were analyzed. The mean length of videos in the experimental and control conditions was 14 min 15 s; the shortest video was 7 min 1 s and the longest was 21 min 15 s. As the part immediately following the start of video recording included filming of setting up and other preparations, analysis was carried out on the 6 min interval from 1 to 7 min after the start of recording.

### Analysis methods

A time sampling method was adopted for behavioral analysis. The 6 min of video were divided into 36 units of 10 s, and each 10 s unit was checked for the following behavioral categories.

(i) Subject talked or made utterance (to PARO/Lion, to staff member, to self, or none).
(ii) Subject touched or stroked PARO/Lion.
(iii) Emotional expression was positive (smiling, laughing), negative (disgust, fear), or neutral.
(iv) Subject laughed.
(v) Staff member talked.

### Evaluators

All video recordings were analyzed by two evaluators with practical training in the range and classification of evaluation behaviors. The behavior evaluation concordance rate between the two evaluators was 95%.

### Evaluate method

The observed frequency of each behavioral category was totaled for the experimental and the control condition for each subject, using the mean values of the two evaluators. Mean observed frequencies of each behavior were calculated separately for M-group and S-group. Observed frequencies of behaviors in the experimental and control condition in M-group and S-group were examined using the Wilcoxon signed-rank test. Statistical processing was carried out using IBM SPSS Statistics 22. Significant level was set at 0.05.

The effect of order of presentation of either PARO or Lion was not counterbalanced in this study. However, the order effect can be disregarded as the S-group scored a mean of 8.8 points and the M-group scored a mean of 16.4 points on Hasegawa’s Dementia Scale, indicating that the subjects are expected to have almost no short-term memory.

## Results

**Figure 2:**
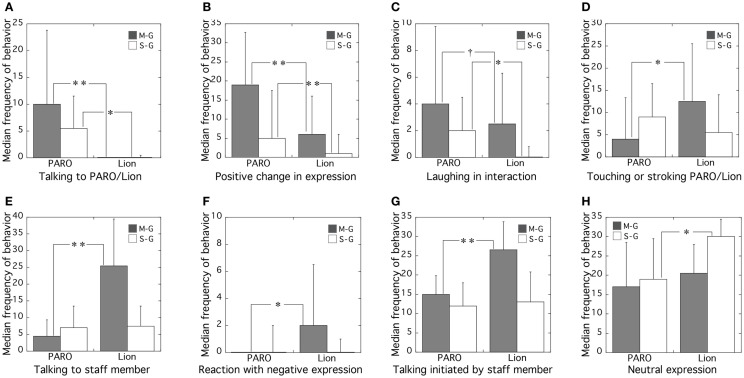
**Median frequencies of observed behaviors of mild/moderate dementia group (M-G) and severe dementia group (S-G) in PARO and Lion conditions**. Observed behaviors were **(A)** talking to PARO/Lion, **(B)** positive change in expression, **(C)** laughing in interaction, **(D)** touching or stroking PARO/Lion, **(E)** talking to staff member, **(F)** reaction with negative expression, **(G)** talking initiated by staff member, and **(H)** neutral expression. Error bars show quartile deviations. (*p < 0.05, **p < 0.01, ^†^0.05 < *p* < 0.1).

Items that were significant in both groups or in one group are shown in Figure 2 and Table 1.

**Table 1:** **Results of statistical analysis (Wilcoxon signed-rank test) of behavioral responses observed in PARO and Lion conditions for mild/moderate dementia group (M-group) and severe dementia group (S-group)**.

	Category of behavior	Mild/moderate dementia group	Severe dementia group	Interpretation
(1) Significant difference in both groups	(A) Talking to PARO/Lion	*T* = 0	*T* = 0	Talking to PARO was more frequent than talking to Lion.
		*N* = 15	*N* = 7	
		*p* < 0.01	*p* < 0.05	
	(B) Positive changes in expression	*T* = 21	*T* = 0	Higher observed frequency of positive expressions when interacting with PARO.
		*N* = 17	*N* = 8	
		*p* < 0.01	*p* < 0.01	
	(C) Laughing with PARO/Lion	*T* = 34	*T* = 0	Higher observed frequency of laughing when interacting with PARO compared to Lion.
		*N* = 16	*N* = 7	
		*p* = 0.078	*p* < 0.05	
(2) Significant difference in M-group only	(D) Touching or stroking PARO/Lion	*T* = 27.5	*T* = 10	Frequency of touching or stroking was higher with Lion than PARO in M-group only.
		*N* = 17	*N* = 7	
		*p* < 0.05	*p* > 0.1	
	(E) Talking to staff member	*T* = 3	*T* = 28	Frequency of talking to staff member was higher with Lion than PARO in M-group only.
		*N* = 18	*N* = 11	
		*p* < 0.01	*p* > 0.1	
	(F) Reaction with negative expression	*T* = 13.5	*T* = 7	Higher observed frequency of negative expressions when interacting with Lion in M-group only. However, the frequency of negative expressions was low in both conditions in both groups.
		*N* = 12	*N* = 5	
		*p* < 0.05	*p* > 0.1	
	(G) Talking initiated by staff member	*T* = 30.5	*T* = 23.5	Higher observed frequency of talking initiated by staff when interacting with Lion in M-group only.
		*N* = 19	*N* = 10	
		*p* < 0.01	*p* > 0.1	
(3) Significant difference in S-group only	(H) Neutral expression	*T* = 57.5	*T* = 8	Higher frequency of neutral expression when interacting with Lion in S-group only.
		*N* = 19	*N* = 10	
		*p* > 0.1	*p* < 0.05	

There were three kinds of significant differences; (1) in both groups, (2) in M-group only, and (3) in S-group only.

(1) Significant differences in both groups had three items: (A) talking to PARO/Lion, (B) positive changes in emotional expression, and (C) laughing Significant with PARO.
(2) Significant differences in M-group only had four items: (D) touching or stroking PARO/Lion, (E) talking to staff member, (F) reaction with negative expression, (G) talking initiated by staff member.
(3) Significant difference in S-group only had only one item as (H) neutral expression.

### Significant differences between responses to PARO and Lion found in both M-group and S-group

In both groups, the frequencies of (A) talking to PARO/Lion and (B) positive changes in emotional expression were significantly higher with PARO than with Lion, and (C) laughing was slightly higher with PARO than with Lion.

There was a strongly significant difference between talking to PARO and talking to Lion in the M-group (*p* < 0.001). There was a marginally significant difference in laughing between PARO and Lion in both M-group (*p* = 0.081) and in S-group (*p* = 0.054).

Females tended to accept both Lion and PARO. Males accepted PARO more than Lion, and the majority of them accepted PARO and cuddled it. In interventions with PARO, 72% of subjects cuddled the PARO, and it was likely that their interest increased in the PARO’s characteristic of not just moving but also actually responding. Even some subjects who disliked animals showed interest in PARO.

### Significant differences between responses to PARO and to Lion found only in M-group

The frequencies of (D) touching and stroking PARO/Lion, (E) talking to the staff member, (F) negative facial expressions, and (G) talking initiated by the staff member were significantly higher with the lion than PARO in the M-group.

There were more people in the M-group who talked to the staff member. When the staff member was with Lion, more subjects talked to the staff member (*p* < 0.001) and talking initiated by the staff member was more common (*p* < 0.01) than with PARO. In other words, the results in the M-group indicated that when PARO was presented to subjects, they spent better time with PARO and less talking to the staff member. However, when Lion was presented, the subjects in M-group demanded more communication with the staff member.

Although the observed frequency of negative expression was low in both groups in both conditions, it was only significantly greater during interaction with Lion in the M-group. This may be linked to the finding that the frequency of talking initiated by the staff member was greater during the interactions with Lion in the M-group. Lion did not have reaction and was not cute for the subjects in M-group.

### Significant difference between responses to PARO and Lion in S-group only

The frequency of neutral expressions was significantly higher with Lion than PARO in S-group only, suggesting that subjects with severe dementia were more affective with PARO than with Lion.

## Conclusion/Discussion

There were studies in which the reactions of elderly people with dementia to PARO were compared to living dog or reading activity as ordinary interventions at elderly facilities (Moyle et al., 2013; Robinson et al., 2013a). However, there were no comparisons between PARO and stuffed toy animals, and between elderly people with mild/moderate dementia (M-group) and those with severe dementia (S-group).

In this study, each interaction of subjects and PARO or Lion was video-taped and coded for the number of times that the subjects looked at, smiled, touched, and talked to and about PARO or Lion. Qualitative analysis was used to code the open-ended questions (Robinson et al., 2013b).

PARO was popular even among people who did not like animals, and its attraction was such that they would spontaneously cuddle it. Moreover, PARO might be able to address the unmet needs of elderly people with severe dementia. Some people refused to interact with PARO or Lion, since at least 1 out of 10 persons usually refuse to interact with these kinds of devices.

Subjects in both groups showed greater frequency of talking to PARO, positive emotional expressions, and a tendency to laugh in their interaction with PARO than in their interaction with Lion. PARO improved their moods very much.

Subjects in the M-group showed a greater frequency of touching or stroking to Lion in order to stimulate it to have reactions. They had negative emotional expressions to Lion though they did not to PARO. They also showed a greater frequency of talking to the staff member. The staff members initiated talking to the subjects in M-group with greater frequency when the subject was with Lion than with PARO.

This result related to loneliness of the subjects. PARO was more accepted by subjects in M-group and they had better time than Lion. In the case of Lion, the subjects in M-group needed to communicate with the staff member more, and expressed their demand by verbally and negative emotional expressions more than those of the subjects in S-group.

As the similar results, in the result of randomized controlled trials at a long-term care facility to compare PARO and a resident dog, it was found that PARO reduced the sense of loneliness significantly (Robinson et al., 2013a). In addition, there were reports that subjects felt less loneliness when they have sessions with pets than those with mechanical dog type robot (Kanamori et al., 2002; Banks et al., 2008). Referring these results, in this study, PARO was closely accepted as like a pet and reduced loneliness more than Lion.

When PARO was presented, it acted as an icebreaker allowing people to initiate conversation. When Lion was presented, there was a greater need for staff to initiate communication with subjects with dementia, especially in M-group.

Subjects in the S-group often talked to PARO as well, but there was no change in the frequency of talking with the staff member. They had higher frequency of neutral expression to Lion than PARO. This means that the subjects in the S-group had much interest in PARO, but did not in the Lion.

In the present study, the first 6 min of behavior when interacting with PARO were analyzed. PARO facilitated the start of conversation of the subjects with dementia and improved their moods. Therefore, intervention with PARO has potential to increase willingness of staff members to communicate and work with elderly people with dementia, especially those with mild/moderate dementia who express their demand of communication more than those with severe dementia.

## Author Contributions

Dr. Kazue Takayanagi carried out the experiment at the facility for elderly people. Dr. Takanori Shibata directed the care using PARO. Dr. Takahiro Kirita was in charge of psychological evaluation.

## Conflict of Interest Statement

Two Robots were offered for free by Dr. Takanori Shibata. The Seiwakai Foundation paid 1,000,000 yen to Iwate Prefectural University for the psychological analysis of the participants’ behavior.
